# Health technology assessment in the Brazilian National Health System: profile of CONITEC exclusion recommendations, 2012-2023

**DOI:** 10.1590/S2237-96222024v33e20240057.en

**Published:** 2024-12-06

**Authors:** Francielli Salles Pinheiro, Stéfani Sousa Borges, Fernanda d’Athayde Rodrigues

**Affiliations:** 1 Universidade Federal do Rio Grande do Sul, Departamento de Produção e Controle de Medicamentos, Rio Grande do Sul, RS, Brazil; 2 Universidade de Brasília, Programa de Pós-Graduação em Saúde Coletiva, Brasília, DF, Brazil

**Keywords:** Evaluación de Tecnologías en Salud, Sistema Único de Salud, Economía de la Salud, Análisis Documental, Health Technology Assessment, Brazilian National Health System, Health Care Economics, Document Analysis

## Abstract

**Objective:**

To analyze the recommendations for exclusion of health technologies in the Brazilian National Health System (SUS), made by the National Commission for the Incorporation of Technologies in the Brazilian National Health System (CONITEC) from 2012 to 2023, and to identify the disinvestment criteria used.

**Methods:**

Documentary, descriptive and retrospective analysis of CONITEC reports that assessed technology exclusion requests.

**Results:**

We identified 24 reports on 74 technologies, whereby the requests predominantly involved medications (95.9%). CONITEC favorably recommended 95% of the exclusions, prioritizing the absence of registration with the National Health Surveillance Agency and the existence of therapeutic alternatives.

**Conclusion:**

Low demand for exclusions compared to incorporations reveals challenges in identifying obsolescence and resistance to exclusion of technologies. The sustainability of the SUS requires greater monitoring of incorporated technologies, to optimize resources and promote the efficiency of the health system.

## Study contributions

**Main results:**

The study identified that demands for exclusion of technologies in the Brazilian National Health System (*Sistema* Único *de* *Saúde* - SUS) are significantly lower than demands for incorporation, highlighting the need to pay greater attention to the disinvestment process, in order to guarantee the sustainability of the health system.

**Implications for services:**

The findings of this study point to the importance of structuring disinvestment assessment methods, with the aim of improving monitoring of health technology obsolescence and promoting more efficient allocation of resources in the SUS.

**Perspectives:**

We suggest that criteria be established for the periodic re-assessment of incorporated technologies, in order to guarantee regular reviews and the fluidity of CONITEC’s work in assessing health technologies.

## RESUMEN

**Objetivo:**

Investigar las solicitudes de exclusión de tecnologías sanitarias en el Sistema Único de Salud (SUS) analizadas por National Committee for Health Technology Incorporation in the Unified Health System (Conitec) de 2012 a 2023, e identificar los criterios de desinversión utilizados.

**Métodos:**

Análisis documental, descriptivo y retrospectivo de los informes de Conitec que evaluaron solicitudes de exclusión de tecnología.

**Resultados:**

se identificaron 24 informes, sobre 74 tecnologías, en los cuales, predominantemente, las solicitudes involucraron medicamentos (95,9%). Conitec recomendó favorablemente el 95% de exclusiones, priorizando la ausencia de registro ante Anvisa y la existencia de alternativas terapéuticas.

**Conclusión:**

La baja demanda de exclusiones en comparación con las incorporaciones revela desafíos en la identificación de obsolescencia y resistencia a deshacerse de tecnologías. La sostenibilidad del SUS requiere un mayor monitoreo de tecnologías incorporadas para optimizar recursos y promocionar la eficiencia del sistema de salud.

## INTRODUCTION

Health systems face resource limitations, requiring careful assessment of investments in new technologies, which include medications, products and procedures used to provide health care to the population.^1^ The rapid advancement of technologies highlights the importance of health technology assessment (HTA) to provide scientific, economic and ethical support in decision-making, whether to incorporate technologies or to exclude them.^2^ HTA is a set of multidisciplinary practices that investigate the clinical, social and economic implications of health technologies, as well as their dissemination and use.^3^

In Brazil, the Brazilian National Health System (*Sistema Único de Saúde* - SUS), in accordance with Law No. 8080/1990,^4^ is based on the principles of universal access to services, comprehensive care and equity. Thus, management of health technologies encompasses assessment, incorporation, dissemination, management and removal of technologies from the health system, considering health needs, the public budget and the responsibilities of different levels of government and social watchdog bodies.^5^

With the aim of expanding the population’s access to technologies, the National Health Surveillance Agency (*Agência Nacional de Vigilância Sanitária* - ANVISA) has been working, since the 2000s, in the field of medication regulation, being responsible for granting health registration for commercialization in Brazil. A decade later, by means of Law No. 12401/2011,^6^ the National Commission for the Incorporation of Technologies in the Brazilian National Health System (*Comissão Nacional de Incorporação de Tecnologias no Sistema Único de Saúde* - CONITEC) was created, forming a legal framework for the use of HTA methods to support the SUS. 

CONITEC is a collegial body responsible for advising the Ministry of Health on the incorporation, exclusion and alteration of medications, products and procedures, creation or alteration of Clinical Protocols and Therapeutic Guidelines (*Protocolos Clínicos e Diretrizes Terapêuticas* - PCDT) and updating the National List of Essential Medications (*Relação Nacional de Medicamentos Essenciais* - RENAME). Its structure is made up of the Medications, Products and Procedures Committees, and Clinical Protocol and Therapeutic Guidelines Committees, in addition to the Executive Secretariat of the Health Ministry.^6^

Requests sent to CONITEC, which can be made by any individual or legal entity, whether or not linked to the SUS, involve an administrative process, with the following steps: 1) Receiving the request and assessing compliance; 2) Initial analysis of the Exclusion Request; 3) Request for additional studies and research, if necessary; 4) Committee analysis and preliminary recommendation; 5) Submission to public consultation and assessment of contributions; 6) Analysis and final recommendation of the Committees; 7) Public hearing, if necessary; 8) Final decision by the Secretary and publication of the ordinance in the Official Gazette of the Union.^7^

The growing production of knowledge about health technologies has generated more requests for inclusion in the SUS, while HTA seeks to select technologies with greater social benefit; although, in view of limited resources, it is crucial to optimize their use. This involves excluding technologies with unfavorable cost-effectiveness and replacing them with more beneficial options, thus improving the sustainable use of available resources.^8^

Different authors have investigated the disinvestment criteria that would be fundamental to promoting greater transparency and facilitating the comprehensive review of all technologies available in health systems. They have agreed that employing methods such as monitoring and evaluating public databases, consulting services and assessing usage trends during specific periods would be relevant approaches for monitoring the performance of technologies and guiding exclusion analyses, according to the real-world context.^9-11^

In this sense, this study aims to analyze the recommendations for the exclusion of health technologies in the SUS, made by CONITEC from 2012 to 2023, identify the disinvestment criteria used and suggest practices that can improve assessment of technology exclusion, in order to achieve more efficient management of SUS resources. 

## METHODS

This is a documentary, descriptive and retrospective analysis of CONITEC recommendations for the exclusion of health technologies, between January 1, 2012 and December 31, 2023. The data were extracted from recommendation reports publicly available on the CONITEC website. As such, it was not necessary to obtain the opinion of a Research Ethics Committee. 

The exclusion reports were identified by means of a manual search in all reports published on the CONITEC website, and relevant information was collected and systematized in a database built using Excel.

In cases in which a report addressed multiple technologies, they were individualized as distinct requests. This strategy was also adopted for different pharmaceutical forms or medication dosages. 

The data extracted from each report included the name of the technology, year of the request, type of technology, indication for exclusion, reason for the request, requesting body and origin of the request (internal or external), final recommendation and justification and, finally, information on therapeutic alternative to meet the request for the technology to be excluded. 

The justifications for exclusion recommendations were analyzed individually and grouped into the following categories: i) Clinical Protocol and Treatment Guidelines (*Protocolos Clínicos e Diretrizes Terapêuticas* - PCDT) changes; National Health Surveillance Agency (*Agência Nacional de Vigilância Sanitária* – ANVISA) registration expired, cancelled or inexistent; iii) Production discontinuation; iv) Existence of available therapeutic alternatives with better therapeutic efficacy and safety profile; v) Clinical evidence of insufficient efficacy; vi) Prohibition of commercialization, distribution, manufacture, import, manipulation and advertising; vii) Exclusion of the technology with which its use was associated; and viii) Disuse of technology in the Specialized Component of Pharmaceutical Care (*Componente Especializado de Assistência Farmacêutica* - CEAF). 

The collected data were analyzed and reported in a descriptive manner and the results were presented in figures and tables, and as absolute and relative frequencies.

## RESULTS

**Figure 1:**
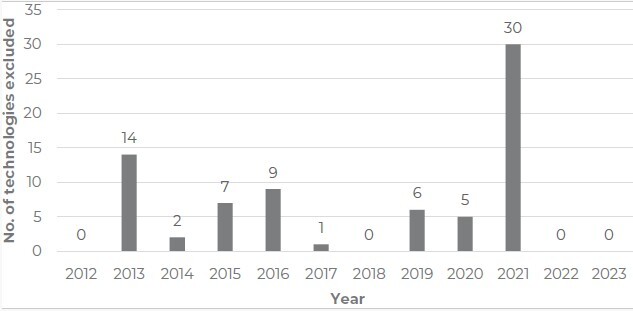
Number of health technologies assessed by CONITEC, from 2012 to 2023, with recommendation for exclusion from the Brazilian National Health System

Over the period from 2012 to 2023, the CONITEC received a total of 1,158 requests,^12^ representing an average of approximately 105 requests per year. Of these, 790 resulted in recommendation reports, with twenty-four reports (3.0%) regarding analysis of recommending exclusion or non-exclusion of technologies. Those twenty-four reports analyzed a total of seventy-four technologies. No requests for exclusion of technologies were recorded in 2012, 2018, 2022 or 2023, while in 2021 thirty technologies were presented with indication for exclusion, twenty-one of which arose from a single report (Figure 1). 

Regarding CONITEC’s final recommendations, we found that 95% of the requests analyzed received a favorable opinion for exclusion. Among those requests, forty-one (58.6%) were fully excluded from the SUS, while the remainder were excluded only for the indications mentioned in the respective reports, and are still available in the SUS for other indications.

Of the total set of requests, 95.9% were related to exclusion of medications, 4.1% were for exclusion of procedures and none were related to exclusion of products. It is important to highlight that all these requests originated from Ministry of Health Departments or Secretariats. Thirty-five requests were submitted by the Ministry of Health’s Science, Technology and Strategic Supplies Secretariat, fourteen by the Health Surveillance Secretariat and six by the Health Care Secretariat. In addition, there were fourteen requests from the Health Care Secretariat’s Department of Specialized Care and five from the Science, Technology and Strategic Supplies Secretariat’s Department of Pharmaceutical Care and Strategic Supplies.

The main justification for exclusion (n = 70), in CONITEC’s final recommendation, was the inexistence of, or canceled or expired ANVISA registration of medication, totaling 31.43% of cases. In 28.57% of them, the justification given was the existence of thrapeutic alternatives with an equivalent efficacy and safety profile also available via SUS for the health condition in question. Other reasons included insufficient clinical evidence of efficacy and disuse of use of the technology in the specialized component (both accounting for 20%). Most reports indicated more than one justification, made by Plenary members, for exclusion, as detailed in Table 1. 

**Table 1:** Justifications for the final CONITEC recommendations found in the health technology exclusion reports, from 2012 to 2023

Justifications for exclusion	Number of technologies (n = 70)	**Percentage of technologies (%)**
PCDT^a^ changes	13	18.57
ANVISA^b^ registration expired, cancelled or inexistent^b^	22	31.43
Production discontinuation	3	4.28
Existence of available therapeutic alternatives with better therapeutic efficacy and safety profile	20	28.57
Clinical evidence of insufficient efficacy	14	20
Prohibition of commercialization, distribution, manufacture, import, manipulation and advertising	3	4.29
Exclusion of the technology with which its use was associated	2	2.86
Disuse of technology in the CEAF^c^	14	20

a) Clinical Protocols and Treatment Guidelines; b) National Health Surveillance Agency; c) Specialized Component of Pharmaceutical Care. Note: Multiple reasons for exclusion were identified for the same technology. The proportion in the third column refers to the total number of individual assessments that with final recommendation for exclusion from the SUS (n = 70).

Among the four requests that received final CONITEC recommendation for non-exclusion in 2021, three shared the same reason, inexistence of current ANVISA registration. The fourth request was made due to the substantially high cost, not justifying its clinical benefits. In these recommendations, CONITEC argued that, despite the medication in question being subject to SUS judicialization, generating a large budgetary impact, it was the only therapeutic alternative available, widely used and of great relevance for SUS health service user treatment.

Just over 85% of the technologies that were excluded were already covered by or had been replaced in the PCDTs, with technologies that were more effective, safer, with better dose convenience, simplified logistics or economically advantageous for the SUS. Seven recommendations for exclusion did not present any available therapeutic alternative (9.46%), three referred to ranitidine hydrochloride in different pharmaceutical forms, subject to a definitive ANVISA ban regarding their commercialization, distribution, manufacture, import, manipulation and advertising, due to the possibility of forming degradation products in the drug molecule. 

Treatment indication with the highest number of requests for technology exclusion was rheumatoid lung disease, with 13 requests (17.57%). Exclusion of nine technologies was requested for hepatitis C, accounting for 12.16% of requests. Treatment for both malaria and Crohn’s disease treatment were both subject to five exclusion requests (6.76%), while there were six requests (8.11%) for antiretroviral therapy. There were four or fewer exclusion requests for each of the remaining treatment indications registered.

## DISCUSSION

Since the establishment of the CONITEC in 2012, the requests for incorporating technologies were, numerically, much higher than those for exclusion. Considering this proportion, and after analyzing the exclusion reports, we were able to verify that there is no standardized structure or specific method for CONITEC assessment, nor did the reports we analyzed consider all relevant aspects of scientific evidence - efficacy, effectiveness and safety ‒ or complete economic studies. Taking the example of other countries, the definition of criteria for reassessing incorporated technologies should guarantee periodic reviews of incorporated technologies and provide greater work fluidity and, consequently, greater sustainability of the SUS.^9,13^

Medications accounted for 73.3% of all requests received by the CONITEC as at 2023, and 95.5% of exclusion requests. Medications represent a significant increase in healthcare expenditure around the world. In Brazil, in 2019, expenditure on medications accounted for 1.8% of the gross domestic product and 18.6% of final expenditure on health goods and services.^14^ Meanwhile, countries such as the United Kingdom, France, Canada, Australia and New Zealand face the challenge of balancing rising medication expenditure within sustainable budgets, especially given the need to fund new, high-cost therapies. There is a growing global trend towards disinvestment in medications in order to create space for new therapies, so that countries can cope with the growth in healthcare expenditure in an equitable and efficient manner.^15^

The were some reasons for excluding health technologies that were not identified in the exclusion recommendation reports we analyzed, such as the acceptance by health professionals and patients for medication formulations providing greater therapeutic convenience. Social and ethical issues that could add long-term efficiency gains to the SUS, such as equity in access, distributive justice and cultural aspects, were also not mentioned.^16,17^

The existence of valid ANVISA registration is a criterion set out in the legal regulations for assessing new technologies,^4^ as well as being a fundamental regulatory tool for assessing medications.^18^ The most cited justification for excluding technologies was the inexistence of registration with ANVISA, or that their registration had expired or been cancelled. CONITEC recommendation Report No. 694^19^ contains a request for the exclusion of 21 medications, requested by the Health Ministry’s Department of Pharmaceutical Care and Strategic Supplies, affirming that their registration had expired or had been canceled in Brazil. Throughout that report, it was stated that hydroxyurea 500 mg capsules, used to treat sickle cell anemia, had valid registration in force in Brazil and was the only medication indicated by the PCDTs as a preventive measure for frequent and severe primary crises and complications and, as such, the Commission’s final recommendation was against exclusion. 

Hydrocortisone cypionate (10 mg and 20 mg tablets) was incorporated into the SUS, in March 2015, for treatment of congenital adrenal hyperplasia, and a review of the PCDTs, including the technology itself, was recommended. After reading the aforementioned documents, we found that the recommended review did not take place. Given the lack of valid registration, its exclusion was requested; however, CONITEC’s final recommendation was against exclusion, as it considered that maintaining the medication’s formulations would be of great relevance to patients, given that the Ministry of Health could purchase and import it in this format. 

The remaining medications covered by Report No. 694 had final CONITEC recommendation in favor of exclusion, and it was possible to note that, in addition to the absence of ANVISA registration, all of them had low consumption and low records or no records of purchases on the Health Prices Database (*Banco de Preços em Saúde*) in recent years. 

A further four medications were excluded on the grounds of lack of ANVISA registration, one of them being injectable molgramostim 300 mg, used in the treatment of aplastic anemia, myelodysplastic syndrome, constitutional neutropenia, HIV-related conditions and bone marrow or pancreas transplantation. Furthermore, lack of commercialization, disuse in the Specialized Component of Pharmaceutical Care (*Componente Especializado de Assistência Farmacêutica*) and existence of an alternative with a better safety profile (injectable filgrastim 300 mg) were cited.^20^

Lack of clinical evidence, one of the pillars of HTA, was the justification for the exclusion of fourteen health technologies throughout the period studied. Examples include the exclusion of the drugs adalimumab, certolizumab, etanercept, infliximab, golimumab, rituximab, abatecept and tocilizumab for treatment of rheumatoid lung disease (ICD M05.1) and rheumatoid vasculitis (ICD M05.2).^21^

Another frequent justification for exclusion was the existence of therapeutic alternatives with better clinical efficacy and safety responses. Telaprevir and boceprevir are antivirals that act directly on structures of the hepatitis C virus and increase the chances of viral negativization.^22^ Both were incorporated into the SUS in July 2012 and included on the RENAME, as part of a set of actions by the Ministry of Health that aimed to review and update the PCDTs for chronic hepatitis C in force at the time. However, one of the important adverse effects of these antivirals is the reduction of blood cells, causing neutropenia, thrombocytopenia and anemia, which should be treated with injectable interferons, made available by the CEAF for this purpose, namely filgrastim (in cases of neutropenia) and epoetin alfa (in cases of anemia). In 2015, new medications for the treatment of chronic hepatitis C were assessed by the CONITEC and incorporated into the SUS – sofosbuvir, daclatasvir and simeprevir –, which proved to be more effective and safe, served a larger number of individuals and had a lower cost for the Ministry of Health, in addition to not requiring the use of injectable interferon. With the inclusion of these drugs on the RENAME and in the PCDT for hepatitis C, the Ministry of Health’s Science, Technology and Strategic Supplies Secretariat requested the exclusion of the drugs boceprevir and telaprevir for use in the SUS, as well as the procedures associated with their use, and use of filgrastim and injectable epoetin alfa, and the CONITEC gave its opinion in favor of exclusion.

Health technology performance assessment refers to the continuous assessment of incorporated technologies, comparing the results achieved in the context of the health system with the expected results agreed to when they were incorporated. Ideally, all health technologies incorporated into the system should constantly undergo performance assessment, as part of the process of health care disinvestment and reinvestment. However, the processes involved in health technologies disinvestment are generally more complex than the initial incorporation decision, as they face challenges such as insufficient scientific evidence, biases in publications, in addition to political, ethical and social issues, since disinvestment can be mistakenly seen as “loss of an acquired right”.^23^ An example of this is resistance to change due to prior clinical training and clinical preferences of prescribers and consumers.^24^

The sustainability of a system can be understood as the ability to promote and maintain positive results over time. In the context of the SUS, sustainability can be influenced by a series of factors, such as adequacy of funding and effectiveness in managing services and products provided.^25^ Therefore, exclusion of technologies is fundamental for the rational allocation of resources intended for public health.^26^

The CONITEC represents a significant advance in HTA in Brazil, being central to decisions on funding and access to pharmaceutical products in the SUS.^27^ However, analysis of the reports highlighted the lack of a standardized structure in assessment of disinvestment, with criteria often focused on absence of ANVISA registration and the availability of therapeutic alternatives. Furthermore, a gap was identified in the consideration of aspects such as acceptance by health professionals, equity in access and cultural aspects, which could add efficiency and sustainability to the SUS. 

The limitations of this analysis include the dependence on data available in CONITEC reports, which may not capture all the nuances of disinvestment recommendations and may not fully reflect the internal dynamics of the assessments made by its Committees. However, it is possible to highlight the need to define clear criteria involving clinical and economic data assessed in the CONITEC HTA process and periodic reviews of incorporated technologies, aligned with international practices, to guarantee efficient management of resources and promote equity in access to innovative treatments on the SUS.
