# Supplementary material for: Health technology assessment in the Brazilian National Health System: profile of CONITEC exclusion recommendations, 2012-2023
Source: Epidemiol Serv Saude. 2024 Dec 6;33:e20240057. doi: 10.1590/S2237-96222024v33e20240057.en (PMC11654042; doi:10.1590/S2237-96222024v33e20240057.en)
Supplement: Translation

# Translation: Avaliação de tecnologias em saúde no Sistema Único de Saúde: perfil das recomendações de exclusão na Conitec, de 2012 a 2023

**DOI:** 10.1590/S2237-96222024v33e20240057.pt

**Authors:** Francielli Salles Pinheiro, Stéfani Sousa Borges, Fernanda d’Athayde Rodrigues

**Affiliations:** 1 Universidade Federal do Rio Grande do Sul, Departamento de Produção e Controle de Medicamentos, Rio Grande do Sul, RS, Brasil; 2 Universidade de Brasília, Programa de Pós-Graduação em Saúde Coletiva, Brasília, DF, Brasil

**Keywords:** Avaliação de Tecnologias em Saúde, Sistema Único de Saúde, Economia da Saúde, Análise Documental

## Abstract

**Objetivo:**

Analisar as recomendações de exclusão de tecnologias em saúde no Sistema Único de Saúde (SUS), feitas pela Comissão Nacional de Incorporação de Tecnologias no Sistema Único de Saúde (Conitec) de 2012 a 2023, e identificar os critérios de desinvestimento utilizados.

**Métodos:**

Análise documental, descritiva e retrospectiva, dos relatórios da Conitec que avaliaram solicitações de exclusão de tecnologias.

**Resultados:**

Foram identificados 24 relatórios, sobre 74 tecnologias, em que, predominantemente, as solicitações envolveram medicamentos (95,9%). A Conitec recomendou favoravelmente 95% das exclusões, priorizando a ausência de registro na Agência Nacional de Vigilância Sanitária e a existência de alternativas terapêuticas.

**Conclusão:**

A baixa demanda de exclusões em comparação às incorporações revela desafios na identificação da obsolescência e resistência à desincorporação de tecnologias. A sustentabilidade do SUS exige maior monitoramento das tecnologias incorporadas, para otimização dos recursos e promoção da eficiência do sistema de saúde.

## Contribuições do estudo

**Principais resultados:**

O estudo identificou que demandas de exclusão de tecnologias no Sistema Único de Saúde (SUS) são significativamente menores que para incorporação, destacando a necessidade de maior atenção ao processo de desinvestimento, para garantia da sustentabilidade do sistema de saúde.

**Implicações para os serviços:**

Os achados deste estudo apontam para a importância de se estruturarem métodos de avaliação de desinvestimento, visando aprimorar o monitoramento da obsolescência de tecnologias em saúde e promover uma alocação mais eficiente de recursos no SUS.

**Perspectivas:**

Sugere-se o estabelecimento de critérios para a reavaliação periódica das tecnologias incorporadas, a fim de serem garantidas revisões regulares e a fluidez do trabalho da Conitec na avaliação de tecnologias em saúde.

## INTRODUÇÃO

Os sistemas de saúde enfrentam limitações de recursos, exigindo uma avaliação criteriosa dos investimentos em novas tecnologias, que incluem medicamentos, produtos e procedimentos usados na prestação de cuidados à saúde da população.^1^ O avanço rápido das tecnologias destaca a importância da avaliação de tecnologias em saúde (ATS) para fornecer subsídios científicos, econômicos e éticos na tomada de decisões, seja para incorporar tecnologias, seja para desincorporar tecnologias.^2^ A ATS é um conjunto de práticas multidisciplinares que investiga as implicações clínicas, sociais e econômicas das tecnologias em saúde, bem como sua difusão e uso.^3^

No Brasil, o Sistema Único de Saúde (SUS), conforme a Lei nº 8.080/1990,^4^ é fundamentado nos princípios da universalidade de acesso aos serviços, integralidade da assistência e equidade. Assim, a gestão de tecnologias em saúde engloba avaliação, incorporação, difusão, gerenciamento e retirada de tecnologias do sistema de saúde, considerando as necessidades de saúde, o orçamento público e as responsabilidades dos diferentes níveis de governo e do controle social.^5^

Com o objetivo de ampliar o acesso da população às tecnologias, a Agência Nacional de Vigilância Sanitária (Anvisa) tem atuado, desde os anos 2000, no campo da regulação de medicamentos, sendo responsável pela concessão de registro sanitário para comercialização no território nacional. Uma década mais tarde, com a Lei nº 12.401/2011,^6^ criou-se a Comissão Nacional de Incorporação de Tecnologias no Sistema Único de Saúde (Conitec), o que representou um marco legal da utilização de métodos de ATS para subsídio ao SUS. 

A Conitec é um órgão colegiado responsável por assessorar o Ministério da Saúde na incorporação, exclusão e alteração de medicamentos, produtos e procedimentos, constituição ou alteração de Protocolos Clínicos e Diretrizes Terapêuticas (PCDTs) e atualização da Relação Nacional de Medicamentos Essenciais (Rename). Sua estrutura compõe-se dos Comitês de Medicamentos, Produtos e Procedimentos, e de Protocolos Clínicos e Diretrizes Terapêuticas, além da Secretaria Executiva.^6^

As solicitações encaminhadas à Conitec, que podem ser feitas por qualquer pessoa física ou jurídica, ligadas ou não ao SUS, envolvem processo administrativo, com as seguintes etapas: 1) Recebimento da solicitação e avaliação de conformidade; 2) Análise inicial da SE; 3) Solicitação de estudos e pesquisas complementares, se necessário; 4) Análise dos comitês e recomendação preliminar; 5) Submissão à consulta pública e avaliação das contribuições; 6) Análise e recomendação final dos comitês; 7) Audiência pública, se necessário; 8) Decisão final do secretário e publicação da portaria no Diário Oficial da União.^7^

A crescente produção de conhecimento sobre tecnologias em saúde tem gerado mais solicitações de inclusão no SUS, enquanto a ATS busca selecionar tecnologias com maior benefício social; mas, diante de recursos limitados, é crucial otimizar seu uso. Isso envolve excluir tecnologias com custo-efetividade desfavorável, em prol de opções mais benéficas, melhorando a utilização sustentável dos recursos disponíveis.^8^

Diferentes autores investigaram os critérios de desinvestimento que seriam fundamentais para promover maior transparência e facilitar a revisão abrangente de todas as tecnologias disponíveis nos sistemas de saúde. Concordaram que empregar métodos como monitoramento e avaliação dos bancos de dados públicos, consulta aos serviços e a avaliação das tendências de uso durante períodos específicos seriam abordagens relevantes para acompanhar o desempenho das tecnologias e orientar análises de exclusão, de acordo com o contexto de mundo real.^9-11^

Nesse sentido, este estudo tem como objetivos analisar as recomendações de exclusão de tecnologias em saúde no SUS, feitas pela Conitec de 2012 a 2023, identificar os critérios de desinvestimento utilizados e sugerir práticas que possam aprimorar a avaliação de desincorporação de tecnologias, para uma gestão mais eficiente dos recursos do SUS. 

## MÉTODOS

Trata-se de análise documental, descritiva e retrospectiva das recomendações de exclusão de tecnologias em saúde na Conitec, entre 1º de janeiro de 2012 e 31 de dezembro de 2023. Os dados foram extraídos dos relatórios de recomendação disponíveis publicamente no sítio eletrônico da Conitec, portanto, não foi necessário parecer de um Comitê de Ética em Pesquisa. 

Os relatórios de exclusão foram identificados por busca manual em todos os relatórios publicados no sítio eletrônico da Conitec, e as informações relevantes foram coletadas e sistematizadas em um banco de dados construído utilizando-se o Excel.

Nos casos em que um relatório abordava múltiplas tecnologias, procedeu-se à individualização delas como demandas distintas. Essa estratégia foi adotada também para diferentes formas farmacêuticas ou dosagens de medicamentos. 

Os dados extraídos de cada relatório incluíram o nome da tecnologia, ano da solicitação, tipo de tecnologia, indicação da exclusão, motivo da solicitação, órgão demandante e origem da demanda (interna ou externa), recomendação final e justificativa e, por fim, informações sobre alternativa terapêutica para suprir a demanda da tecnologia a ser excluída. 

As justificativas para as recomendações de exclusão foram analisadas individualmente e agrupadas nas categorias: i) Mudanças nos Protocolos Clínicos e PCDTs; ii) Registro expirado, cancelado ou ausente na Anvisa; iii) Descontinuação da produção; iv) Existência de alternativas terapêuticas disponíveis com melhor perfil terapêutico de eficácia e segurança; v) Evidências clínicas de eficácia insuficientes; vi) Proibição da comercialização, distribuição, fabricação, importação, manipulação e propaganda; vii) Exclusão da tecnologia à qual seu uso era associado; e viii) Desuso da tecnologia no Componente Especializado de Assistência Farmacêutica (CEAF). 

Os dados coletados foram analisados e reportados de forma descritiva e os resultados apresentados em figuras e tabelas, e na configuração de frequências absolutas e relativas.

## RESULTADOS

Ao longo do período de 2012 a 2023, a Conitec recebeu um total de 1.158 demandas,^12^ uma média de aproximadamente 105 demandas por ano. Dessas, 790 resultaram em relatórios de recomendação, sendo 24 relatórios (3,0%) referentes à análise de recomendação de exclusão ou não exclusão de tecnologias. Nesses 24 relatórios, um total de 74 tecnologias foram analisadas. Não foi registrada nenhuma solicitação de exclusão de tecnologias em 2012, 2018, 2022 e 2023, enquanto em 2021 foram apresentadas 30 tecnologias com indicação para exclusão, 21 provenientes de um único relatório (Figura 1). 

**Figura 1:** Número de tecnologias em saúde avaliadas pela Conitec, nos anos de 2012 a 2023, que tiveram recomendação de exclusão do Sistema Único de Saúde

No que se refere à recomendação final da Conitec, constatou-se que 95% das demandas analisadas receberam parecer favorável para a exclusão. Entre essas demandas, 41 (58,6%) foram integralmente excluídas no âmbito do SUS, enquanto as demais foram excluídas apenas para as indicações mencionadas nos respectivos relatórios, estando ainda disponíveis no SUS para outras indicações.

Do conjunto total de solicitações, 95,9% estavam relacionadas à exclusão de medicamentos, 4,1% versavam sobre a exclusão de procedimentos e nenhuma era referente à exclusão de produtos. Importante ressaltar que todas essas demandas se originaram de departamentos ou secretarias do Ministério da Saúde. Foram submetidas 35 demandas pela Secretaria de Ciência, Tecnologia e Insumos Estratégicos do Ministério da Saúde (SCTIE/MS), 14 pela Secretaria de Vigilância em Saúde (SVS/MS) e seis pela Secretaria de Atenção à Saúde (SAS/MS). Além disso, houve 14 demandas do Departamento de Atenção Especializada da SAS (DAE/SAS/MS) e cinco do Departamento de Assistência Farmacêutica e Insumos Estratégicos da SCTIE (DAF/SCTIE/MS).

A principal justificativa para exclusão (n = 70), na recomendação final da Conitec, foi a ausência de registro do medicamento na Anvisa, registro cancelado ou expirado, totalizando 31,43% dos casos. Em 28,57%, a justificativa foi a existência de alternativas terapêuticas com perfil de eficácia e segurança equivalentes já disponíveis no SUS para a indicação. Outras razões incluíram a insuficiência de evidências clínicas de eficácia e o desuso da tecnologia no componente especializado (ambas representando 20%). A maioria dos relatórios indicaram mais de uma justificativa, pelos membros do Plenário, para a exclusão, conforme detalhado na Tabela 1. 

**Tabela 1:** Justificativas referentes à recomendação final da Conitec presentes nos relatórios de exclusão de tecnologias em saúde, nos anos de 2012 a 2023

Justificativas para a exclusão	Número de tecnologias (n = 70)	**Porcentagem de tecnologias (%)**
Mudanças nos PCDTs^a^	13	18,57
Registro expirado, cancelado ou ausente na Anvisa^b^	22	31,43
Descontinuação na produção	3	4,28
Existência de alternativas terapêuticas com melhor perfil terapêutico de eficácia e segurança	20	28,57
Evidências clínicas de eficácia insuficiente	14	20
Proibição da comercialização, distribuição, fabricação, importação, manipulação e propaganda	3	4,29
Exclusão da tecnologia à qual seu uso era associado	2	2,86
Desuso da tecnologia no CEAF^c^	14	20

a) Protocolos Clínicos e Diretrizes Terapêuticas; b) Agência Nacional de Vigilância Sanitária; c) Componente Especializado da Assistência Farmacêutica. Nota: Múltiplos motivos de exclusão foram identificados para uma mesma tecnologia. A proporção na terceira coluna refere-se ao número total de avaliações individuais que tiveram recomendação final de exclusão do SUS (n = 70).

Entre as quatro solicitações que receberam recomendação final de não exclusão pela Conitec, em 2021, três compartilharam o mesmo motivo, a ausência de registro sanitário vigente. A quarta solicitação teve como motivação o custo substancialmente elevado, não justificando seus benefícios clínicos. Nessas recomendações, a Conitec argumentou que, apesar de o medicamento em questão ser objeto de judicialização no SUS, gerando um grande impacto orçamentário, se tratava de única alternativa terapêutica disponível, amplamente utilizada e de grande relevância para o tratamento dos pacientes.

Pouco mais de 85% das tecnologias que foram excluídas já eram cobertas ou foram substituídas nos PCDTs, por tecnologias mais eficazes, seguras, de melhor conveniência posológica, logística simplificada ou economicamente vantajosas para o SUS. Sete recomendações de desincorporação não apresentavam nenhuma alternativa terapêutica disponível (9,46%), e três referiam-se ao cloridrato de ranitidina em diferentes apresentações farmacêuticas, objeto de proibição definitiva pela Anvisa quanto a sua comercialização, distribuição, fabricação, importação, manipulação e propaganda, devido à possibilidade de formar produtos de degradação da própria molécula. 

A indicação terapêutica com maior número de solicitações para exclusão de tecnologias foi a doença reumatoide de pulmão, com 13 pedidos (17,57%). Para hepatite C, foi solicitada a desincorporação de nove tecnologias, representando 12,16% das solicitações. Tanto o tratamento de malária quanto da doença de Crohn foram objeto de cinco solicitações de exclusão cada (6,76%), enquanto terapia antirretroviral recebeu seis solicitações (8,11%). As demais indicações registraram quatro ou menos solicitações de exclusão cada.

## DISCUSSÃO

Desde o estabelecimento da Conitec, em 2012, as demandas de incorporação de tecnologias foram, em números, muito superiores às de desincorporação. Considerando essa proporção, e após análise dos relatórios de exclusão, foi possível constatar que não há uma estrutura padronizada ou um método específico na avaliação adotada pela Conitec, tampouco os relatórios avaliados consideraram todos os aspectos relevantes sobre a evidência científica ‒ eficácia, efetividade e segurança ‒ e estudos econômicos completos. A exemplo de outros países, a definição de critérios para reavaliação de tecnologias incorporadas garantiria revisões periódicas das tecnologias incorporadas e proporcionaria maior fluidez do trabalho, consequentemente, maior sustentabilidade do SUS.^9,13^

Os medicamentos representaram 73,3% de todas as demandas recebidas na Conitec até o ano de 2023, e 95,5% das solicitações de exclusão. Medicamentos representam significativo aumento dos gastos em saúde em todo o mundo. No Brasil, em 2019, as despesas com medicamentos representaram 1,8% do produto interno bruto e 18,6% dos gastos finais de bens e serviços em saúde.^14^ Enquanto isso, países como Reino Unido, França, Canadá, Austrália e Nova Zelândia enfrentam o desafio de equilibrar o aumento dos gastos com medicamentos dentro de orçamentos sustentáveis, especialmente diante da necessidade de financiar novas terapias de alto custo. Há uma tendência global crescente para o desinvestimento em medicamentos, a fim de se criar espaço para novas terapias, de modo que os países possam lidar com o crescimento dos gastos em saúde de maneira equitativa e eficiente.^15^

Algumas motivações para exclusão de tecnologias em saúde não foram identificadas nos relatórios de recomendação de exclusão analisados, como a aceitação dos profissionais de saúde e pacientes por apresentações de maior comodidade terapêutica. Questões sociais e éticas que poderiam agregar ganhos de eficiência a longo prazo para o SUS, como equidade no acesso, justiça distributiva e aspectos culturais, também não foram citadas.^16,17^

A existência de registro válido na Anvisa é um critério disposto no regimento legal para avaliação de novas tecnologias,^4^ bem como uma ferramenta regulatória fundamental na avaliação de medicamentos.^18^ A justificativa para exclusão de tecnologias mais apontada foi a ausência de registro sanitário na Anvisa, ou seu registro expirado ou cancelado. O relatório de recomendação da Conitec nº 694^19^ conta com a solicitação de exclusão de 21 medicamentos, demandada pelo DAF/SCTIE/MS, cujos registros sanitários estariam expirados ou cancelados no Brasil. Ao longo do relatório, foi constatado que a hidroxiureia em cápsulas de 500 mg, utilizada para o tratamento de anemia falciforme, possuía registro válido vigente no Brasil e era o único medicamento indicado no PCDT como preventivo de crises e complicações primárias frequentes e severas, e por isso teve recomendação final contrária à exclusão. 

O medicamento cipionato de hidrocortisona (em comprimidos de 10 mg e 20 mg) foi incorporado ao SUS, em março de 2015, para o tratamento de hiperplasia adrenal congênita, e recomendou-se a revisão do PCDT, incluindo a tecnologia. Realizando-se a leitura dos referidos documentos, verificou-se que a atualização recomendada não ocorreu. Dada a inexistência de registro válido, foi solicitada sua exclusão; porém, a recomendação final da Conitec foi contrária à desincorporação, por considerar que a manutenção das apresentações do medicamento seria de grande relevância para os pacientes, visto que ele poderia ser adquirido via importação pelo Ministério da Saúde. 

Os demais medicamentos presentes no relatório nº 694 tiveram recomendação final da Conitec favoráveis à desincorporação, e foi possível observar que, além da ausência de registro sanitário, todos apontavam baixo consumo e baixo ou nenhum registro de compra no Banco de Preços em Saúde nos últimos anos. 

Outros quatro medicamentos foram excluídos sob a justificativa de ausência de registro sanitário, sendo um deles o molgramostim 300 mg injetável, utilizado no tratamento de anemia aplásica, mielodisplásica, neutropenias constitucionais, doenças pelo HIV e transplante de medula ou pâncreas. Além disso, foi observada a ausência de comercialização, desuso no CEAF e a existência alternativa com melhor perfil de segurança (filgrastima 300 mg injetável).^20^

A falta de evidências clínicas, um dos pilares da ATS, foi a justificativa para a exclusão de 14 tecnologias em saúde ao longo do período estudado. Como exemplos, temos a exclusão dos medicamentos adalimumabe, certolizumabe, etanercepte, infliximabe, golimumabe, rituximabe, abatecepte e tocilizumabe para o tratamento da doença reumatoide do pulmão (CID M05.1) e vasculite reumatoide (CID M05.2).^21^

Outra justificativa frequente de exclusão foi a existência de alternativas terapêuticas com melhor resposta clínica de eficácia e segurança. Telaprevir e boceprevir são antivirais que agem diretamente em estruturas do vírus da hepatite C e aumentam as chances de negativação viral.^22^ Ambos foram incorporados no SUS em julho de 2012 e incluídos na Rename, como parte de um conjunto de ações do Ministério da Saúde que visavam à reforma e atualização do PCDT da hepatite C crônica vigente à época. Porém, um dos efeitos adversos importantes desses antivirais é a diminuição de células do sangue, causando neutropenia, trombocitopenia e anemia, que deveriam ser tratadas com interferons injetáveis, disponibilizados pelo CEAF para essa finalidade, a filgrastima (nos casos de neutropenia) e a alfaepoetina (nos casos de anemia). Em 2015, foram avaliados pela Conitec e incorporados novos medicamentos para o tratamento da hepatite C crônica no SUS – sofosbuvir, daclastavir e simperevir –, que demonstraram ser mais eficazes e seguros, atendiam a uma parcela maior de indivíduos e possuíam menor custo para o Ministério da Saúde, além de não exigirem o uso de interferon injetável. Com a inclusão desses medicamentos na Rename e no PCDT da hepatite C, a SCTIE/MS solicitou a exclusão dos medicamentos boceprevir e telaprevir do SUS, bem como os procedimentos associados ao uso deles, e o uso de filgrastima e alfaepoetina injetável, com parecer da Conitec favorável à exclusão.

A avaliação do desempenho das tecnologias em saúde (AdTS) refere-se à avaliação continuada das tecnologias incorporadas, comparando os resultados alcançados no contexto do sistema de saúde às expectativas de resultados acordados na incorporação. Idealmente, todas as tecnologias incorporadas ao sistema deveriam passar constantemente pela AdTS, para o processo de desinvestimento e reinvestimento em saúde. Porém, as modalidades de desinvestimento de tecnologias em saúde geralmente se apresentam mais complexas que a decisão inicial de incorporação, por enfrentarem desafios como a insuficiência de evidências científicas, vieses em publicações, além de questões políticas, éticas e sociais, uma vez que a ação de desinvestimento pode ser entendida, de forma equivocada, como “perda de um direito adquirido”.^23^ Um exemplo disso é a resistência à mudança devido ao treinamento clínico já estabelecido e a preferências clínicas dos prescritores e do consumidor.^24^

A sustentabilidade de um sistema pode ser compreendida como a habilidade de promover e manter resultados positivos ao longo do tempo. No contexto do SUS, a sustentabilidade pode ser influenciada por uma série de fatores, como a adequação do financiamento e a eficácia na gestão dos serviços e produtos disponibilizados.^25^ Dessa forma, a exclusão de tecnologias é fundamental para a alocação racional de recursos destinados à saúde pública.^26^

A Conitec representa um avanço significativo na ATS no Brasil, sendo central na decisão do financiamento e acesso a produtos farmacêuticos no SUS.^27^ No entanto, a análise dos relatórios evidenciou a falta de uma estrutura padronizada na avaliação de desinvestimento, com critérios frequentemente focados na ausência de registro sanitário e na disponibilidade de alternativas terapêuticas. Além disso, identificou-se uma lacuna na consideração de aspectos como aceitação pelos profissionais de saúde, equidade no acesso e aspectos culturais, que poderiam agregar eficiência e sustentabilidade ao SUS. 

As limitações desta análise incluem a dependência de dados disponíveis nos relatórios da Conitec, que podem não captar todas as nuances das recomendações de desinvestimento e não refletir completamente as dinâmicas internas das avaliações dos seus Comitês. No entanto, é possível destacar a necessidade da definição de critérios claros envolvendo dados clínicos e econômicos avaliados no processo de ATS da Conitec e revisões periódicas das tecnologias incorporadas, alinhadas com práticas internacionais, para se garantir uma gestão eficiente dos recursos e promover a equidade no acesso a tratamentos inovadores no SUS.
